# Junctional E-cadherin/p120-catenin Is Correlated with the Absence of Supporting Cells to Hair Cells Conversion in Postnatal Mice Cochleae

**DOI:** 10.3389/fnmol.2018.00020

**Published:** 2018-02-21

**Authors:** Wen-wei Luo, Xin-wei Wang, Rui Ma, Fang-lu Chi, Ping Chen, Ning Cong, Yu-yan Gu, Dong-dong Ren, Juan-mei Yang

**Affiliations:** ^1^Department of Otology and Skull Base Surgery, Eye & ENT Hospital of Fudan University, Shanghai, China; ^2^Key Laboratory of Hearing Medicine, Ministry of Health, Shanghai, China; ^3^Department of Cell Biology, Emory University, Atlanta, GA, United States

**Keywords:** cochlea, supporting cells, hair cells, SC-to-HC conversion, DAPT, E-cadherin/p120ctn complexes

## Abstract

Notch inhibition is known to generate supernumerary hair cells (HCs) at the expense of supporting cells (SCs) in the mammalian inner ear. However, inhibition of Notch activity becomes progressively less effective at inducing SC-to-HC conversion in the postnatal cochlea and balance organs as the animal ages. It has been suggested that the SC-to-HC conversion capacity is inversely correlated with E-cadherin accumulation in postnatal mammalian utricles. However, whether E-cadherin localization is linked to the SC-to-HC conversion capacity in the mammalian inner ear is poorly understood. In the present study, we treated cochleae from postnatal day 0 (P0) with the Notch signaling inhibitor DAPT and observed apparent SC-to-HC conversion along with E-cadherin/p120ctn disruption in the sensory region. In addition, the SC-to-HC conversion capacity and E-cadherin/p120ctn disorganization were robust in the apex but decreased toward the base. We further demonstrated that the ability to regenerate HCs and the disruption of E-cadherin/p120ctn concomitantly decreased with age and ceased at P7, even after extended DAPT treatments. This timing is consistent with E-cadherin/p120ctn accumulation in the postnatal cochleae. These results suggest that the decreasing capacity of SCs to transdifferentiate into HCs correlates with E-cadherin/p120ctn localization in the postnatal cochleae, which might account for the absence of SC-to-HC conversion in the mammalian cochlea.

## Introduction

Sensory hair cells (HCs) within the inner ear play a key role in converting mechanical stimuli to neuronal signals, which is important for both auditory and vestibular functions. HC loss caused by noise exposure, infection, toxicity, or aging in mammals is irreversible due to lack of ability to produce new HCs (Forge et al., 1993; Warchol et al., 1993; Kelley et al., 1995; Kawamoto et al., 2009). Recent studies have shown that the mice neonatal inner ear SCs have limited ability to regenerate HCs (Chai et al., 2012; Jan et al., 2013; Cox et al., 2014; Wang et al., 2015; Chen et al., 2017; Lu et al., 2017); however the adult mice cochlea loss this ability. In contrast, in the ears of some lower vertebrates, such as sharks, bony fish, zebra fish, amphibians, reptiles, and birds, supporting cells (SCs) can give rise to regenerated HCs throughout life to rapidly restore sensory function (Corwin and Cotanche, 1988; Ryals and Rubel, 1988; He et al., 2015, 2017).

The underlying reason why mammalian SCs cannot regenerate HCs has been studied for many years by investigating the differences between the epithelia of the inner ear in mammals and non-mammals (Hackett et al., 2002; Davies et al., 2007; Meyers and Corwin, 2007; Burns et al., 2008, 2013; Lu and Corwin, 2008; Collado et al., 2011a,b; Burns and Corwin, 2013, 2014; Cheng et al., 2017; Zhang et al., 2017). It has been demonstrated that F-actin bands and E-cadherin at apical SC-SC junctions in fish, amphibians, chickens and birds remain thin throughout life, whereas their counterparts grow much thicker in mice and humans as they mature postnatally (Burns et al., 2008, 2013; Burns and Corwin, 2014). Intercellular junctions and their actin bands in the epithelia of non-mammalian species play pivotal roles in regulating growth and renewal such that cell death and extrusion are matched by cell replacement (Ingber, 2008; Miyoshi and Takai, 2008; Cavey and Lecuit, 2009; Meng and Takeichi, 2009; Boggiano and Fehon, 2012; Guillot and Lecuit, 2013). Reinforcement of junctions in mammalian balance organs occurs contemporaneously with decreases in SC proliferation and differentiation (Davies et al., 2007; Meyers and Corwin, 2007; Collado et al., 2011a), which play an important role in restricting HC replacement in the mammalian vestibular system (Burns et al., 2008, 2013; Collado et al., 2011b; Burns and Corwin, 2014).

Application of the γ-secretase inhibitor DAPT, which is a Notch signaling inhibitor, leads to HC regeneration through both direct differentiation and mitotic generation of SCs in the mammalian cochleae (Xiao et al., 2003; Collado et al., 2011b; Li et al., 2015, 2016; Maass et al., 2015; Wang et al., 2015; Ni et al., 2016a,b; Waqas et al., 2016b). Inhibition of γ-secretase activity becomes progressively less effective at inducing SC-to-HC conversion in the postnatal cochlea and balance organs as the animal ages (Xiao et al., 2003; Collado et al., 2011b; Cox et al., 2014; Li et al., 2015; Maass et al., 2015; Ni et al., 2016a,b; Wu et al., 2016). Previous studies have demonstrated that the DAPT-induced SC-to-HC phenotype conversion capacity is inversely correlated with E-cadherin accumulation in postnatal mammalian utricles (Collado et al., 2011b). However, it is unclear whether E-cadherin/p120ctn localization is correlated with the SC-to-HC conversion capacity in postnatal mammalian cochleae.

E-cadherin and its associated cytoplasmic catenins are responsible for mediating cell-cell adhesion (Steinberg, 2007; Ishiyama et al., 2010; Van den Bossche et al., 2012). In cochlear epithelia, E-cadherin expression is limited to cells in the outer HC (OHC) region and the region lateral to OHCs (Etournay et al., 2010; Chacon-Heszele et al., 2012; Burns et al., 2013). p120ctn is critical for the surface stability of E-cadherin (Ishiyama et al., 2010) and is widely expressed in the sensory epithelium of the cochlea (Chacon-Heszele et al., 2012).

To determine whether E-cadherin/p120ctn localization is correlated with the SC-to-HC conversion in postnatal mammalian cochleae, we generated supernumerary HCs in cultured postnatal mice cochleae at different ages by DAPT treatment. We observed an apparent increase in HC numbers and a significant decrease in SC numbers along with disruption of E-cadherin/p120ctn localization in the sensory region following DAPT treatment. We further demonstrated that the SC-to-HC conversion capacity and E-cadherin/p120ctn disruption decreased with age, consistent with the pattern observed for E-cadherin/p120ctn accumulation in the postnatal cochleae. Moreover, SC-to-HC differentiation competency and E-cadherin/p120ctn disruption displayed regional specificity: these effects were robustly observed in the cochlea apex and limited in the base. This study suggests that E-cadherin/p120ctn localization in the postnatal cochleae plays an important role in stabilizing SC/HC arrangements and limiting SC-to-HC conversion.

## Methods

### Animals

This study was performed in accordance with the recommendations of the Institutional Animal Care and Use Committee of Fudan University. The protocol was approved by the Institutional Animal Care and Use Committee of Fudan University and was in compliance with the NIH guidelines for the care and use of laboratory animals. Postnatal day (P)0, P3, and P7 C57BL/6 mice were purchased from Shanghai SLAC Laboratory Animal Co., Ltd. (Shanghai, China).

### Organotypic culture of neonatal mouse cochleae

Mice were euthanized by carbon dioxide asphyxiation and decapitated. Their heads were placed in 75% ethanol and quickly transferred to chilled Hanks' balanced salt solution (HBSS, HyClone, Logan, UT, USA). The temporal bones were dissected, and the bullae were isolated from the temporal bone using sterile procedures in ice-cold HBSS. The bone and spiral ligament were gently removed with forceps.

Cochlear basilar membrane explants were isolated, seeded intact on glass coverslips coated with Poly-L-Lysine (P4832, Sigma-Aldrich, St. Louis, MO, USA) and maintained in four-well culture dishes (Greiner Bio-One, Frickenhausen, Germany) in Dulbecco's Modified Eagle's Medium/nutrient mixture F-12 (DMEM/F12, Invitrogen, Waltham, MA, USA) containing 2% B27 supplement (Invitrogen, cat. no. 17504044) and 50 μg/ml penicillin (Sigma-Aldrich, St. Louis, MO, USA). A whole cochlear explant is cut into four parts: apex, apex-middle, middle-basal and basal turns (Figure **2A**).

### DAPT and EdU treatment of cochleae cultures

Explant cultures were treated with DAPT (EMD Millipore, cat. no. 565784, Burlington, MA, USA). DAPT was initially dissolved in sterile dimethyl sulfoxide (DMSO, Sigma-Aldrich) to a concentration of 10 mM and stored at 4°C. The 10-mM stock solution was diluted with culture medium to the final working concentration immediately before use. The cultured cochleae were treated with 5 μM DAPT for the entire culture period. Control cochleae were treated with 0.2% DMSO for the time indicated in the results. 5-Ethynyl-2′-deoxyuridine (EdU, RiboBio, Guangzhou, China) to a final concentration of 10 μM was added to the cultured media throughout the entire culture period to label proliferative cells. The tissues were incubated at 37°C in a humidified atmosphere of 95% air and 5% CO_2_, and the medium was changed daily throughout the culture period.

### Immunohistochemistry

Explant cultures were harvested and fixed with 4% paraformaldehyde for 30 min and then treated with 0.1% Triton X-100 plus 10% donkey serum for 1 h. The explants were then incubated with the following primary antibodies for 24 h at 4°C: rabbit anti-myosin7A (1:100; Proteus Biosciences, Ramona, CA, USA), mouse anti-myosin7A (1:200; Developmental Studies Hybridoma Bank, Iowa City, IA, USA), rabbit anti-Prox1 (1:1,000; Millipore), mouse anti E-cadherin (against the C-terminus, 1:200, BD Transduction Laboratories, San Jose, CA, USA), and goat anti-p120-catenin (1:200; Santa Cruz Biotechnology, Dallas, TX, USA). The explants were washed three to five times in PBS and incubated with secondary antibodies overnight at 4°C in the dark. The secondary antibodies included donkey anti-mouse/rabbit/goat Alexa Fluor 555 (1:1,000), donkey anti-mouse/rabbit Alexa Fluor 488 (1:1,000) and/or donkey anti-mouse/rabbit (H+L) Alexa Fluor 647 (1:1,000; Jackson ImmunoResearch, West Grove, PA, USA). EdU staining was performed as described previously (Zhang et al., 2012), and immunofluorescence staining was performed immediately following EdU staining.

### RNA extraction and qRT-PCR

The RNA from two to three pure sensory epithelia of apex and mid-apex cochlear explants was purified from each group using an RNeasy Plus Micro kit (Qiagen, cat. no. 74034, Hilden, Germany) 48 h after DAPT/DMSO treatment. cDNA was synthesized using PrimerScript RT Master mix (RR036A; Takara Bio, Inc., Otsu, Japan) according to the manufacturer's instructions. PCR primers were designed with Primer3 according to the gene sequences obtained from GenBank (http://www.ncbi.nlm.nih.gov/genbank/). β-Actin was used as an endogenous reference. The primer sequences were as follows: β-actin, (F) tctttgcagctccttcgttg, (R) tccttctgacccattcccac; Atoh1, (F) tatctgctgcattctcccga, (R) gctgttcccgtactttgacg; Hes1, (F) cgagcgtgttggggaaatac, (R) cgttgatctgggtcatgcag; Hes5, (F) gaaacacagcaaagccttcg, (R) cgctggaagtggtaaagcag; E-cadherin (F) gtgaagggacggtcaacaac, (R) acagtaggagcagcaggatc; and p120-catenin, (F) tctactccctctgtggtcca, (R) gtcagcttctcaaactgggc.

Real-time qRT-PCR was performed in duplicate using a PreMix SYBR Green kit (TaKaRa, cat. no. RR420A) on a 7500HT Fast Real-Time PCR System (Applied Biosystems, Foster City, CA, USA). PCR cycling consisted of 35 cycles of denaturation for 30 s at 94°C, annealing for 30 s at 57°C, and extension for 30 s at 72°C and a final extension for 5 min at 72°C. All real-time qRT-PCR reactions were performed in triplicate, and the relative quantification of gene expression was analyzed using the 2^−ΔΔ*CT*^ method with the housekeeping gene β-actin as the endogenous reference.

### Western blotting

Proteins were extracted from five pure sensory epithelia of apex and mid-apex cochlear explants that were isolated by removing the surrounding non-sensory epithelium. Western blotting was performed as described previously (Lu and Corwin, 2008). The following antibodies were used: mouse anti-E-cadherin (BD Biosciences, 1:2,500 dilution), anti-P120-catenin (Santa Cruz, 1:500 dilution), and mouse anti-GAPDH (Beyotime, China, 1:1,000 dilution). Proteins were detected using the Image Quant LAS 1040 detection system (GE Healthcare, Piscataway, NJ, USA). The band intensity was measured and normalized against the intensity of the GAPDH band measured from the same lane using ImageJ.

### Image acquisition and cell counts

Fluorescent images were acquired using a Leica SP8 confocal microscope. All of the images were digitally processed using ImageJ and Adobe Photoshop CS5. Images were acquired with a pixel size of 0.035 × 0.035 × 0.30 μm following Nyquist sampling with no pixel saturation to ensure that no structural information was lost. All samples with E-cadherin/p120ctn staining were imaged with the same confocal intensity. The cell counts from the confocal images were performed using Adobe Photoshop CS5. The total number of Myo7a^+^ HCs and Prox1^+^ SCs were quantified from two randomly selected 100-μm regions per specimen along the length of the cochlea in the apical, mid-apical, mid-basal and basal turns. Each group included at least three different cochleae.

### Measurement of p120ctn depletion width and apical junctional regions (AJRs)

The p120ctn depletion width in the sensory region was measured by the lateral-to-medial distance in the SC layer perpendicular to the length of the cochlea. At least three random areas from the apex to the base were analyzed in each sample using ImageJ. The AJR width was measured as the perpendicular distance across the adherens junction and circumferential p120ctn in two adjacent cells that shared a junction, as described previously (Burns et al., 2008). The widths of horizontal AJRs were measured along the length of the cochlea from three random regions per specimen, and each group consisted of at least three different cochleae.

### Statistics

Statistical analyses were conducted using Microsoft Excel, GraphPad Prism 6.0, and SPSS software. A two-tailed, unpaired Student's *t*-test was used to determine the statistical significance between two groups, and one-way ANOVA and post-ANOVA analysis (LSD) were used to determine the statistical significance among three or more groups. All data are presented as the means ± standard errors. Significance was defined as *p* < 0.05.

## Results

### Junctional E-cadherin/p120ctn complexes in the postnatal mouse cochleae increase during maturation

**Figure 1:**
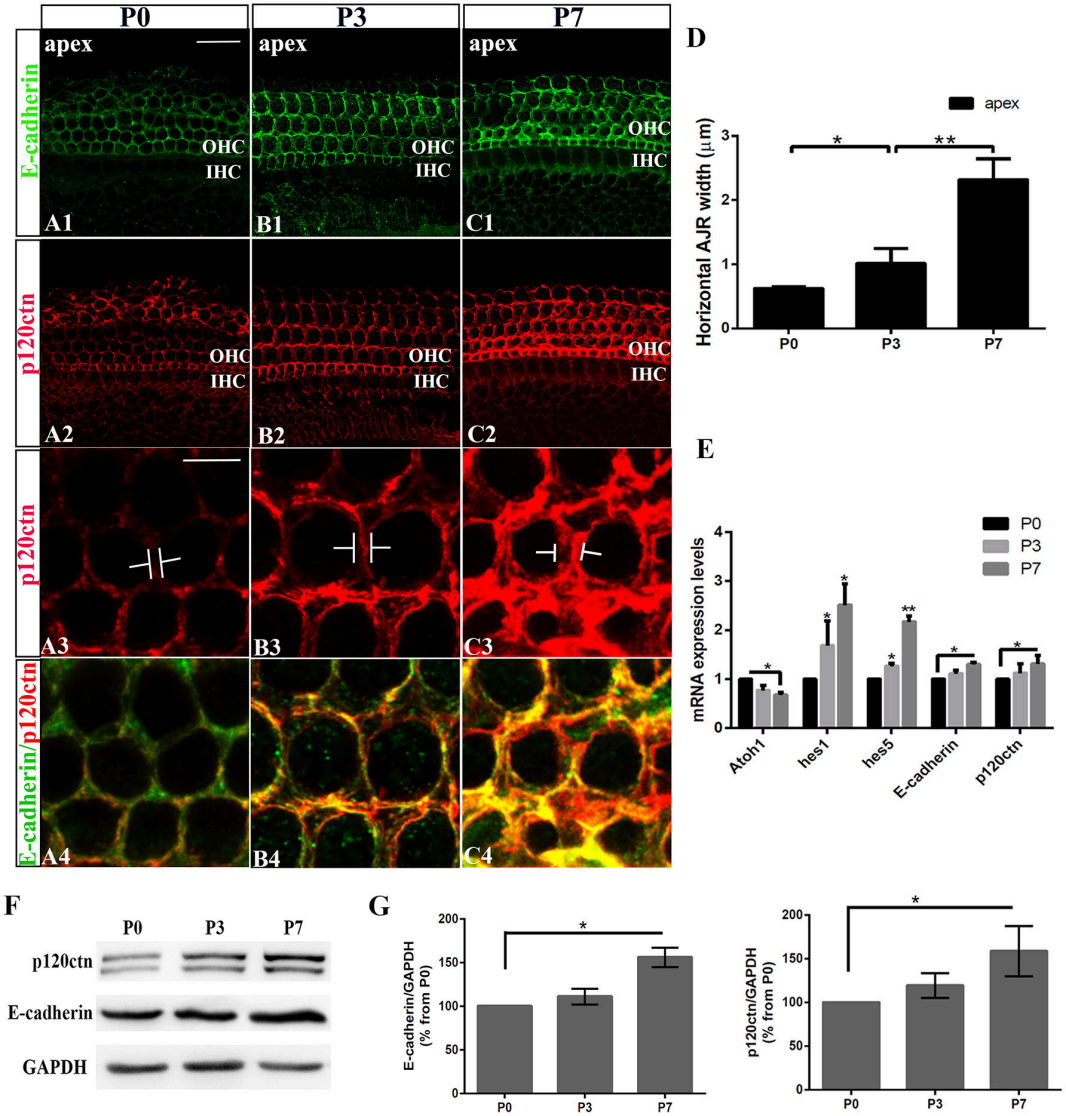
Increase in junctional E-cadherin/p120ctn in the postnatal mice cochleae. **(A–C)** Representative images of the apex turn in cochleae from P1, P3, and P7 mice immuno-labeled for E-cadherin (green) and p120ctn (red) with the same confocal intensity. **(A3–C3)** Magnified images of A2-C2 showing the differences in the junctional p120ctn widths between adjacent outer hair cells in the same row. **(A4–C4)** Double-labeling of E-cadherin (green) and p120ctn (red). (**D)** Quantification of apical junctional region (AJR) widths in the apexes of cochleae from P0, P3, and P7 mice. **(E)** Relative *Atoh1, Hes1, Hes5, E-cadherin*, and *P120-catenin* mRNA expression levels in cochleae from P1, P3, and P7 mice (*n* = 3 for each age). The mRNA levels for each gene were plotted relative to the respective P0 mRNA levels. **(F)** Representative examples of Western blots showing the E-cadherin, p120ctn, and total GAPDH (internal control) protein expression levels in pure cochlear sensory epithelia harvested from P1, P3, and P7 mice. **(G)** Quantification of Western-blot experimental results. E-cadherin and p120ctn were normalized to the total GAPDH levels, and the values are expressed as percentages relative to the P0 levels for comparison. The average percentages relative to P0 are shown (*n* = 3). OHC, Outer hair cell region; IHC, Inner hair cell region. The error bars in **(D,E,G)** show the SEMs. ^*^p < 0.05, ^**^p < 0.01. The scale bars represent 20 μm in **(A1)** and 5 μm in **(A3)**.

We first examined the E-cadherin and p120ctn distribution in the postnatal cochleae from P0, P3, and P7 mice (Figures 1A–C). E-cadherin/ p120ctn were confined to the intercellular junctions of the OHC region (Figures 1A1,B1,C1). The E-cadherin and p120ctn fluorescence intensity markedly increased at cell junctions during the week after birth (Figures 1A–C). We measured the width of the AJRs in the apex region of mice cochleae at P0, P3, and P7, as defined by p120ctn staining. Our results revealed wider AJRs in the apex region at P7 compared with P3 (2.32 ± 0.33 vs. 1.02 ± 0.23 μm, *p* < 0.01; *n* = 4 and 5, respectively) and at P3 compared with P0 (1.02 ± 0.23 vs. 0.62 ± 0.03 μm, *p* < 0.05; *n* = 5 and 4, respectively; Figure 1D). Quantitative RT-PCR revealed higher *Hes1, Hes5, E-cadherin*, and *p120ctn* expression levels and lower *Atoh1* levels in cochleae at P7 compared with P0 (*p* < 0.05; Figure 1E). Both proteins were detectable at P0 but were more intense at P7 (Figure 1F). The relative immunoblot band intensities for total E-cadherin and p120ctn proteins in cochlear sensory epithelia showed a significant change between P0 and P7 (Figure 1G). The total E-cadherin levels increased by 156% (156 ± 11.30%, *n* = 3, *p* < 0.05; Figure 1G) from P0 to P7, and the total p120ctn levels increased by 158% (158 ± 28.68%, *n* = 3, *p* < 0.05; Figure 1G) from P0 to P7. Our results indicate that E-cadherin/p120ctn complexes significantly increase in the postnatal mouse cochlea as the mouse ages.

### Notch inhibition-induced SC-to-HC conversion is primarily restricted to the outer hair cell region

**Figure 2:**
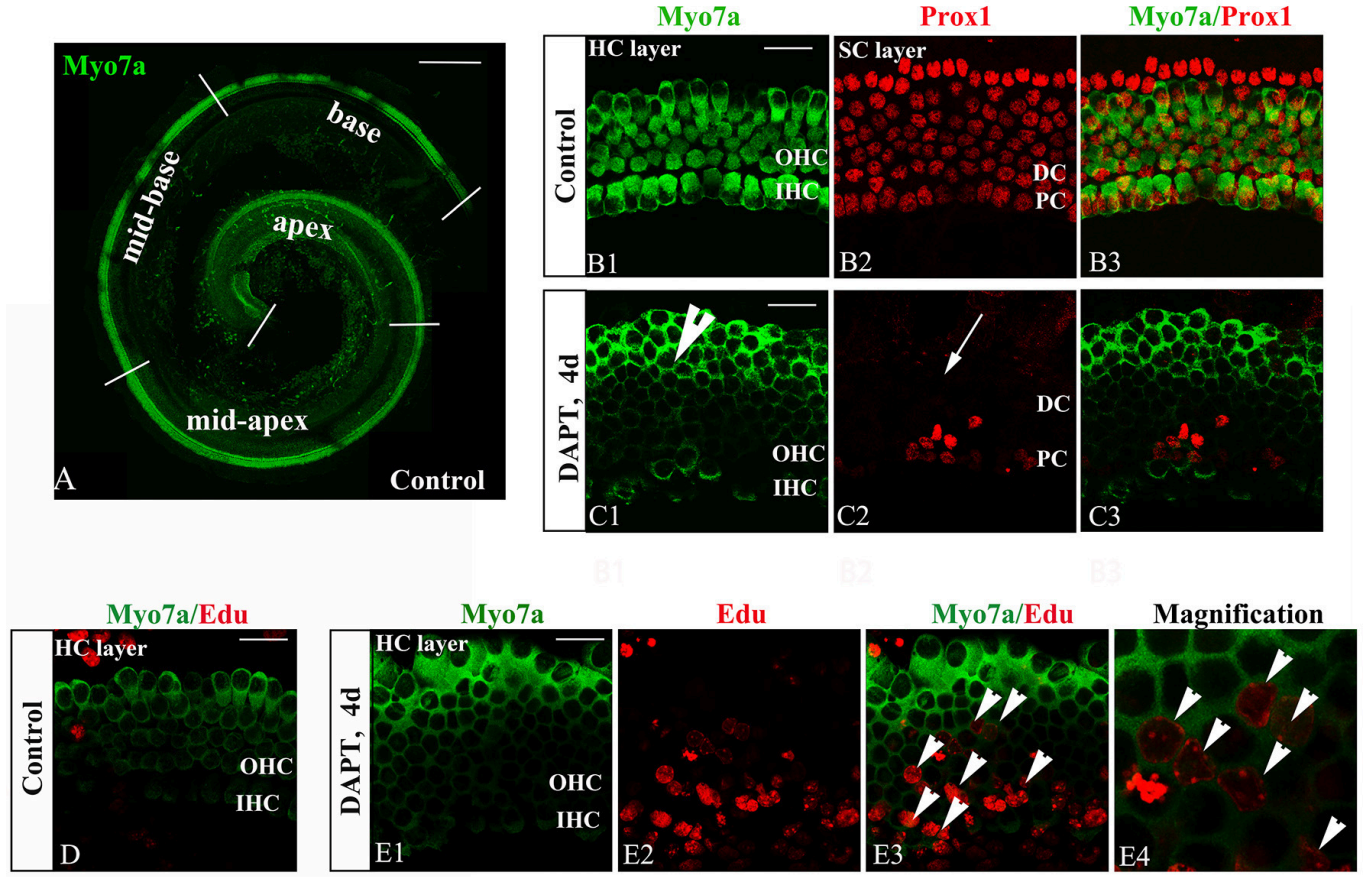
DAPT-induced direct differentiation and mitotic generation of HCs are primarily restricted to the outer hair cell region. **(A)** A postnatal day 0 (P0) organ of Corti was cultured for 24 h. Cochlear explants were divided into the apex, mid-apex, mid-base, and base turn. Myo7a-labeled HCs (green) are shown. **(B,C)** After 4 days of DAPT treatment, numerous Myo7a^+^ HCs (green) were detected in the outer hair cell region, whereas only a few Prox1^+^ SCs (red) were preserved in the pillar cell region of the SC layer compared with the vehicle control. **(D)** In control cochleae, no EdU^+^/Myo7a^+^ HCs were detected in the sensory region of the HC layer. **(E)** In DAPT-treated cochleae, several EdU^+^/Myo7a^+^ HCs were observed in the HC layer. **(E4)** Magnified images of **(E3)**. The explants presented in **(B–E)** were of the apex of P0 cochleae. OHC, Outer hair cell region; IHC, Inner hair cell region; DC, Deiter cell region; PC, Pillar cell region. The scale bars represent 200 μm in **(A)** and 25 μm in **(B–E)**.

The inner ear sensory epithelium consists of both HCs and SCs, and their specification is mediated by lateral inhibition through the Notch signaling pathway. Loss of Notch signaling generates supernumerary HCs at the expense of SCs. Notch inhibition can induce SC-to-HC differentiation through both direct differentiation and mitotic generation of SCs in neonatal mouse cochleae (Li et al., 2015; Ni et al., 2016a). We confirmed these experiments using cultured P0 mouse organs of Corti in the presence of DAPT (Figures 2C,E). DMSO treatment served as a control (Figures 2B,D). DAPT application to cultured cochlear explants for 4 days notably increased the number of Myo7a^+^ HCs and significantly decreased the number of Prox1^+^ SCs in the OHC region (HCs, Figure 2C1, short arrow; SCs, Figure 2C2, long arrow) compared with that found in the control DMSO-treated cochleae (Figure 2B2). To further examine the mitotic generation of HCs *in vitro*, we treated the cultures with EdU throughout the experiment and observed several EdU^+^/Myo7a^+^ HCs 4 days after DAPT treatment (Figure 2E, short arrows). In contrast, we did not observe any EdU incorporation (red) in Myo7a^+^ HCs (green) within the control sensory epithelium (Figure 2D). Thus, Notch inhibition in the organ of Corti resulted in direct SC-to-HC transdifferentiation or mitotic HC generation (Xiao et al., 2003; Li et al., 2015; Ni et al., 2016a,b), which is primarily restricted to the outer hair cell region.

### Notch inhibition disrupts E-cadherin/p120ctn localization in the outer hair cell region of neonatal cochleae

**Figure 3:**
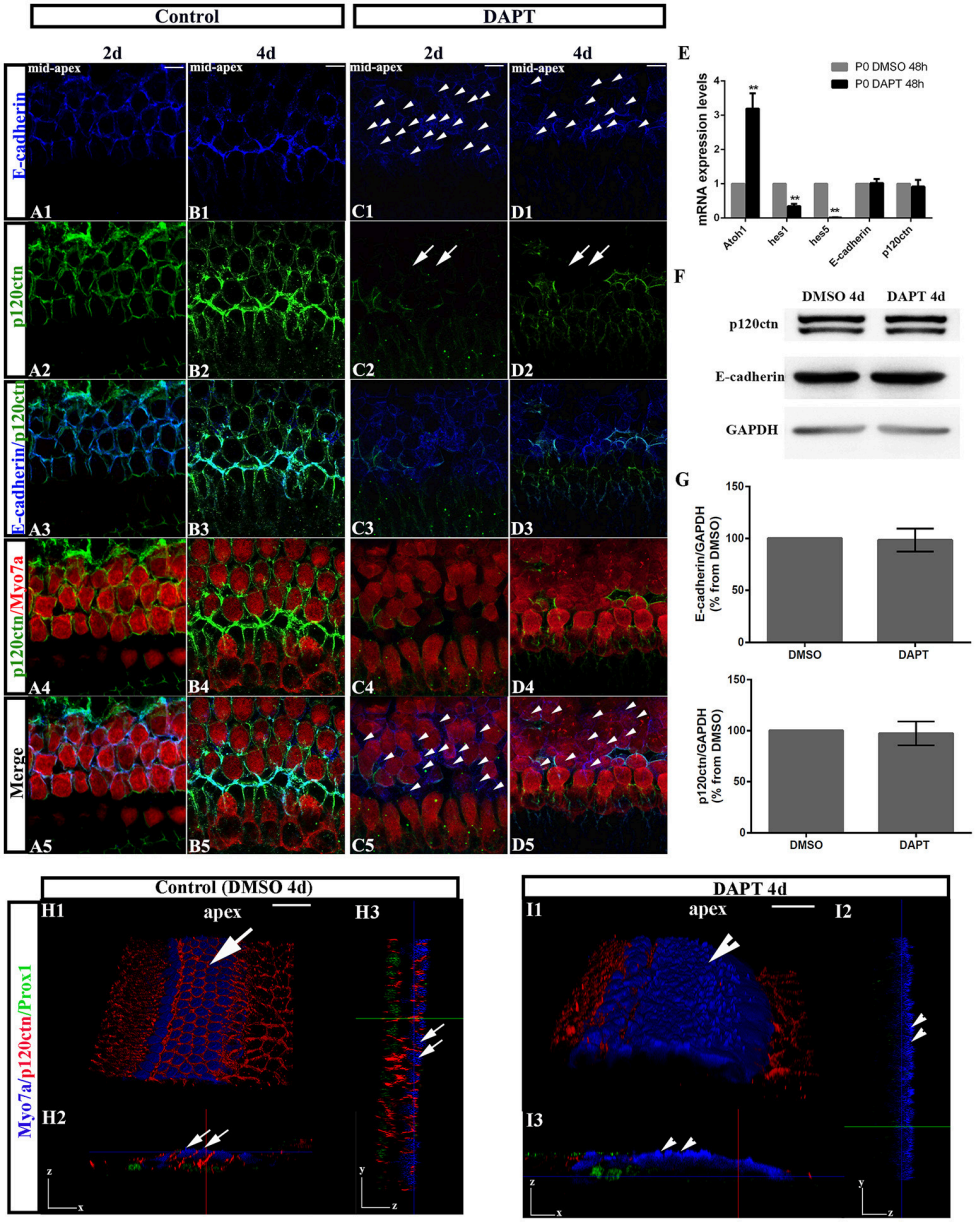
DAPT induces E-cadherin/p120ctn disorganization in the outer hair cell region of the sensory epithelium. **(A–D)** Images of the mid-apex of P0 cochleae treated with DMSO or DAPT for 2–4 days and immunostained for E-cadherin (blue), p120ctn (green), and Myo7a (red). In control cochleae, E-cadherin internalization and p120ctn depletion did not occur in the sensory epithelium **(A,B)**. In the DAPT-treated groups, internalization of E-cadherin occurred along with depletion of p120ctn in the outer hair cell region **(C,D)**. The short arrows in both **(C1,D1)** show that E-cadherin internalization occurred following the DAPT treatments. The long arrows in **(C2)** indicate that p120ctn was decreased in the sensory epithelium after 2 days of DAPT treatment. Following 4 days of DAPT treatment, p120ctn was depleted in the outer hair cell region (long arrows in **D2**). The short arrows in **(C5)** indicate SCs that internalized E-cadherin. The short arrows in **(D5)** represent HCs that internalized E-cadherin. **(E)** Relative *Atoh1, Hes1, Hes5, E-cadherin*, and *p120ctn* mRNA expression levels in P0 cochleae treated with DMSO or DAPT for 48 h (*n* = 3 for each gene). The mRNA levels for each gene were plotted relative to the respective vehicle control mRNA levels. **(F)** Representative images of Western blots showing the E-cadherin, p120ctn, and total GAPDH (internal control) protein expression levels in cochlear sensory epithelia treated with DMSO or DAPT for 4 days. **(G)** Quantification of Western blot experiments. E-cadherin and p120ctn were normalized to the total GAPDH levels, and the values are expressed as percentages relative to DMSO for comparison. Average percentages relative to DMSO are shown (*n* = 3). **(H)** 3D projections showing that Myo7a^+^ HCs and Prox1^+^ SCs in the sensory region are surrounded by p120ctn and do not undergo SC-to-HC conversion in control cochleae (long arrows). HCs and SCs were surrounded by p120ctn in *x-z-*axial sections **(H2)** and in *y-z-*axial sections **(H3)**. **(I)** In DAPT-treated cochleae, p120ctn was largely depleted in both the HC and SC layers, whereas Myo7a^+^ HCs were significantly increased, and Prox1^+^ SCs were dramatically decreased (short arrows). **(I2)** *x-z-*axial sections. **(I3)** *y-z-*axial sections. Myo7a (blue), Prox1 (green), and p120ctn (red). The error bars in **(E,G)** show the SEMs. ^**^p < 0.01. The scale bars represent 10 μm in **(A–D)** and 25 μm in **(H,I)**.

To test whether E-cadherin/p120ctn complexes are involved in SC-to-HC differentiation in the mammalian cochleae, we analyzed their cellular localization through immunofluorescence and imaged the cochleae using the same confocal intensities. The expression patterns of E-cadherin and p120ctn in the OHC region markedly changed after DAPT treatment (Figures 3C,D). In control cochlea, E-cadherin and p120ctn were organized in the cytomembrane of HCs and SCs (Figures 3A,B). Two days after DAPT induction, we observed less E-cadherin at cell-cell contacts and more E-cadherin diffusely distributed in the cytosol of Myo7a^−^ cells (Figures 3C1,C5, short arrows), suggesting that E-cadherin failed to localize to the cytomembrane of SC contacts and was instead endocytosed to the cytoplasm after Notch inhibition. Along with E-cadherin internalization, p120ctn was strikingly decreased at the cytomembranes of both Myo7a^+^ and Myo7a^−^ cells in the OHC region (Figure 3C2, long arrows). Four days after DAPT treatment, p120ctn was completely lost from the membrane (Figure 3D2, long arrows). We observed decreased numbers of Myo7a^−^ cells and increased numbers of Myo7a^+^ cells with internalized E-cadherin (Figure 3D5) compared with two days DAPT application (Figure 3C5). 3D projections showed that HCs and SCs in control cochleae were surrounded by p120ctn in *x-z-*axial sections (Figure 3H2) and in *y-z-*axial sections (Figure 3H3). In DAPT-treated cochleae, p120ctn was largely depleted in both the HC and SC layers in *x-z-*axial sections (Figure 3I2) and in *y-z-*axial sections (Figure 3I3), whereas the number of Myo7a^+^ HCs significantly increased, and the number of Prox1^+^ SCs notably decreased (Figure 3I). These results support previous models in which junctional E-cadherin/p120ctn is correlated with Notch-induced SC-to-HC conversion in the mammalian cochleae.

To investigate whether DAPT treatments inhibit the Notch pathway because they induce cochlear SCs to differentiate into HCs, we first compared the mRNA levels of *Hes1, Hes5*, and *Atoh1* in cochleae cultured for 48 h with DAPT or DMSO through quantitative RT-PCR (Figure 3E). The *Atoh1* mRNA levels in DAPT-treated cochleae were 2.8- to 3.6-fold higher than those in matched controls (*p* < 0.01). In addition, the *Hes1* and *Hes5* mRNA levels notably decreased in DAPT-treated cochleae compared with the control group (*p* < 0.01). These results confirmed that DAPT induces supernumerary HCs by inhibiting Notch signaling and increasing Atoh1, which is consistent with previous results (Collado et al., 2011b; Li et al., 2015; Maass et al., 2015). We also compared the *E-cadherin* and *p120ctn* mRNA levels between cochleae treated with DAPT and DMSO for 48 h (Figure 3E). However, there were no significant changes in the *E-cadherin* and *p120ctn* transcript levels between DAPT-treated cochleae and controls (*p* > 0.05). We also compared protein expression between DAPT- and DMSO-treated cochleae (Figures 3F,G). After 4 days of treatment, Western blotting revealed that the E-cadherin and p120ctn protein levels were similar in the control and DAPT-treated cochleae (Figure 3F). The relative immunoblot band intensities for total E-cadherin and p120ctn protein in the cochlear sensory epithelia did not show significant changes between the control and DAPT-treated groups (*p* > 0.05; Figure 3G).

### Notch inhibition-induced SC-to-HC conversion and p120ctn disorganization are age-dependent

**Figure 4:**
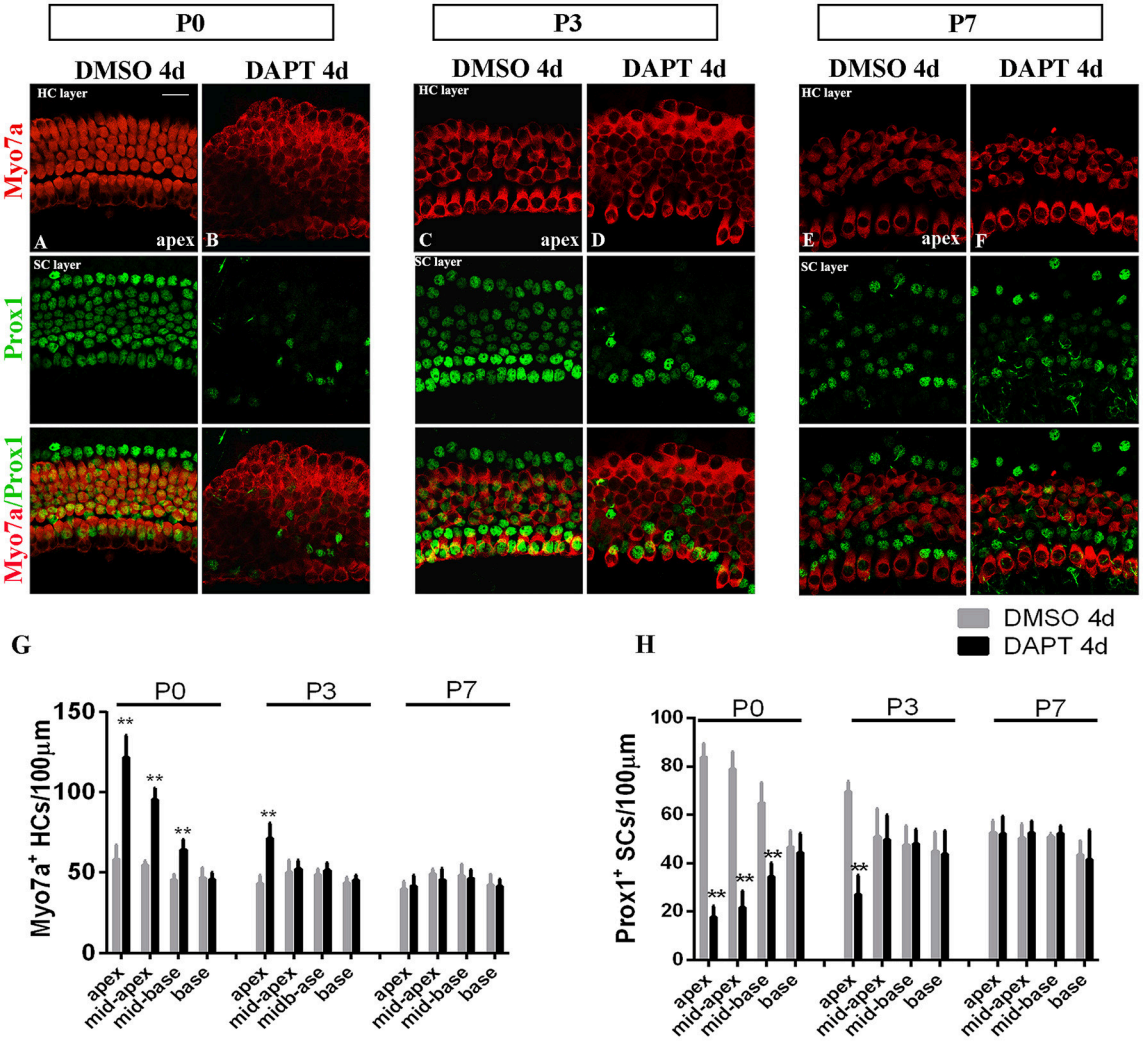
The SC-to-HC conversion capacity differs by region and decreases with age. **(A–F)** The apexes of P0, P3, and P7 cochleae cultured for 4 days in the presence of DAPT or DMSO. **(A,B)** In the apexes of DAPT-treated P0 cochleae, the number of Myo7a^+^ HCs significantly increased and the number of Prox1^+^ SCs significantly decreased compared with the control. **(C,D)** In DAPT-treated P3 cochlea, there was a significant increase in Myo7a^+^ HCs and a decrease in Prox1^+^ SCs compared with DMSO treatment. **(E,F)** In the apexes of P7 cochlea, there were no differences in the numbers of Myo7a^+^ HCs and Prox1^+^ cells between the DAPT treatment group and the control group. **(G,H)** Quantification of the number of Myo7a^+^ HCs/100 μm and Prox1^+^ SCs/100 μm after 4 days of DAPT or DMSO treatments from the apex to the base of cochleae from P0, P3, and P7 mice. The error bars in **(G,H)** show the SEMs. ^**^p < 0.01. The scale bar represents 20 μm.

**Figure 5:**
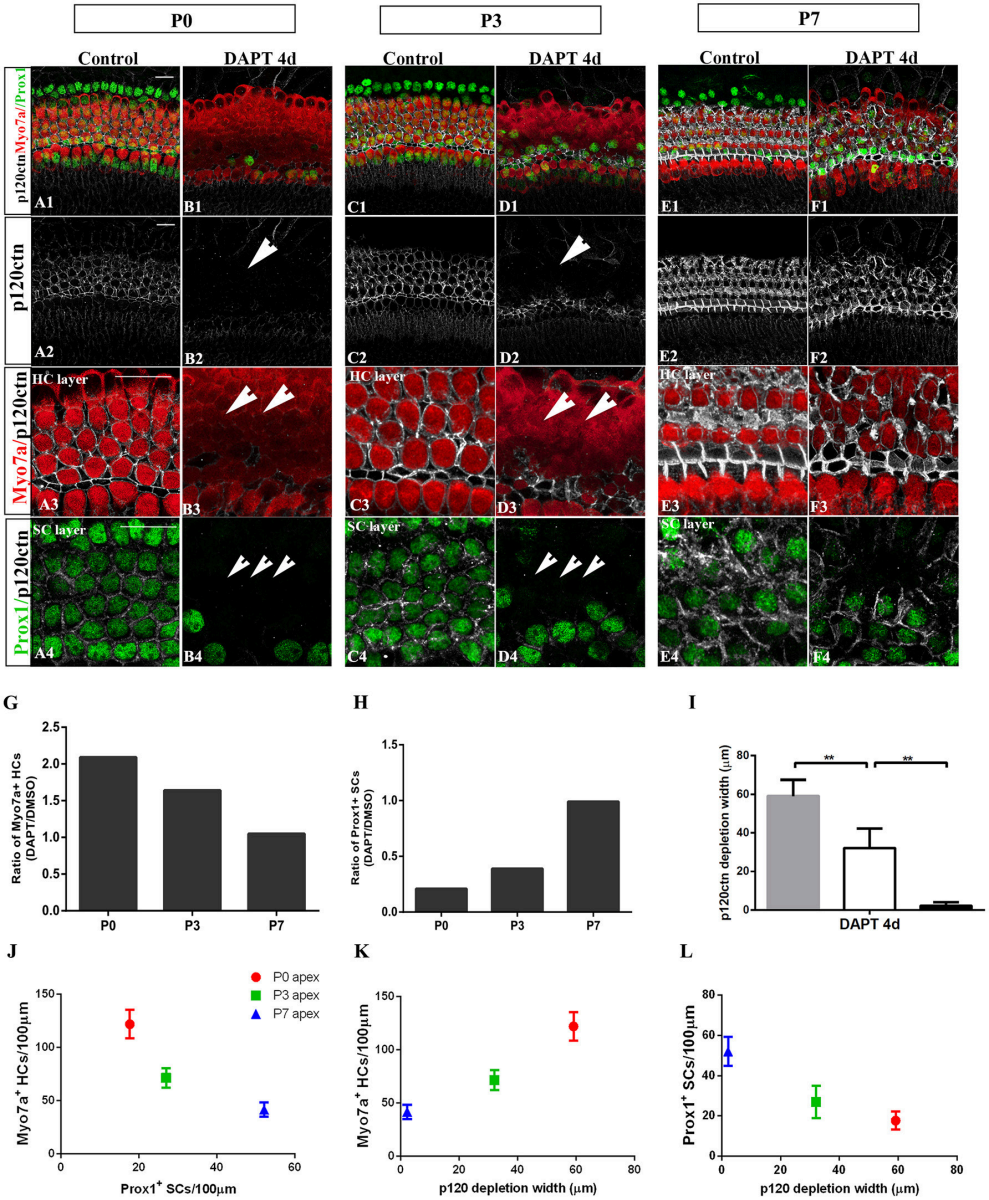
E-cadherin/p120ctn disorganization and SC-to-HC conversion decline with age and cease by P7. **(A–F)** Whole mounts of apexes harvested from P0, P3, and P7 cochleae treated with DMSO and DAPT for 4 days. Myo7a (red), Prox1 (green), and p120ctn (white). p120ctn was imaged with the same confocal intensity for all samples. **(A2–F2)** p120ctn expression in the apex from P0 to P7. p120ctn disintegration was detected in the apexes of P0 (short arrow in **B2**) and P3 (short arrow in **D2**) mice treated with DAPT. No p120ctn disruption was observed in P7 mice or controls. **(A3–F3)** Higher magnification of p120ctn in the HC layers. p120ctn was depleted and HCs were regenerated in P0 (short arrows in **B3**) and P3 mice (short arrows in **F3**). **(A4–F4)** Higher magnification of p120ctn and Prox1^+^ SCs in the SC layer. After DAPT treatments, p120-depleted areas were notably smaller in the apexes of P3 mice (short arrows in **F4**) compared with those in the apexes of P0 mice (short arrows in **B4**). **(G,H)** Ratio of Myo7a^+^ HCs and Prox1^+^ SCs following DAPT treatments compared with DMSO treatments in the apexes of P0, P3, and P7 cochleae. **(G–I)** Quantification of p120ctn depletion widths in the apexes of P0, P3, and P7 mice treated with DAPT for 4 days. **(J)** Scatter plot of the number of Myo7a^+^ HCs/100 μm (*y-*axis) and Prox1^+^ SCs/100 μm (*x-*axis) in the apexes of P0, P3, and P7 mice. A higher number of generated Myo7a^+^ HCs was associated with the retention of fewer Prox1^+^ SCs. **(K,L)** Scatter plot of Myo7a^+^ HCs/100 μm (**I**, y-axis), Prox1^+^ SCs/100 μm (**J**, y-axis), and p120 depletion widths (*x*-axis) in the apexes of P0, P3, and P7 mice. The data in **(G–L)** are presented as the means ± SEMs. ^**^p < 0.01. The scale bars represent 25 μm.

The efficiency of SC-to-HC transdifferentiation in the cochleae and utricles is progressively reduced and eventually ceases with age (Chai et al., 2011; Collado et al., 2011b; Kelly et al., 2012; Liu et al., 2012; Cox et al., 2014; Gao et al., 2016). In this study, we cultured cochlear explants from P0, P3, and P7 mice for 4 days in DAPT and DMSO media (Figures 4, 5). In the apex regions of P0 cochleae, the DAPT-treated group contained two-fold more Myo7a^+^ HCs than the control group (121.93 ± 13.41 vs. 58.30 ± 9.03, *p* < 0.01; *n* = 14 and 14, respectively; Figures 4G, 5G). The number of Prox1^+^ SCs in the DAPT-treated group decreased 79% compared with the DMSO-treated cochleae (17.70 ± 4.47 vs. 83.75 ± 5.83, *p* < 0.01; *n* = 11 and 12, respectively; Figures 4H, 5H). In the apex regions of P3 cochleae, the DAPT-treated group contained 1.6-fold higher numbers of Myo7a^+^ HCs compared with the controls (71.40 ± 9.29 vs. 43.33 ± 5.03, *p* < 0.01; *n* = 5 and 3, respectively; Figures 4G, 5G), whereas the number of Prox1^+^ SCs in the DAPT-treated group decreased by 60% compared with the DMSO-treated cochleae (27.00 ± 7.95 vs. 69.50 ± 4.20, *p* < 0.01; *n* = 6 and 4, respectively; Figures 4H, 5H). However, in the mid-apex to the base of the cochlea at P3 and in the apex to the base at P7, there were no significant differences in the number of Myo7a^+^ HCs or Prox1^+^ SCs between the DAPT group and the controls (Figures 4G,H, 5G,H; *p* > 0.05). We did not observe any signs of phenotypic conversion in any of the four cochlear turns from P7 mice, even after culturing for 4 days with 10 μm DAPT (data not shown). These results provide evidence that inhibition of γ-secretase activity becomes progressively less effective at inducing the SC-to-HC conversion in the postnatal cochlea as the animal ages.

To determine whether E-cadherin/p120ctn disruption is also age-dependent as it is in utricles (Collado et al., 2011b), we cultured the apex regions from P0, P3, and P7 mice in DMSO and DAPT for 4 days (Figures 5A–F). Through fixed confocal intensity, we confirmed that p120ctn depletion declined with age following DAPT induction (Figures 5B,D,F). SCs from the apex of P0 and P3 mice completely lost junctional p120ctn. SCs were converted into HCs in the presence of DAPT (Figures 5B4,D4, short arrows). Additionally, the regions where p120ctn was depleted and where SCs were converted into HCs were much narrower in P3 mice compared with P0 mice (32.11 ± 10.19 vs. 59.12 ± 8.39 μm, *p* < 0.01; *n* = 5 and 5, respectively; Figures 5B,D,I,L). SCs in P7 cochleae retained junctional p120ctn and failed to convert into HCs after continuous DAPT treatment (Figure 5D). We analyzed the association of p120ctn depletion with the number of Myo7a^+^ HCs and Prox1^+^ SCs at P0, P3, and P7. Our results revealed that greater p120ctn depletion from junctions was associated with increased generation of Myo7a^+^ HCs and less preservation of Prox1^+^ SCs (Figures 5J–L). Our results demonstrate that the HC regeneration ability and E-cadherin/p120ctn disruption decreased synchronously with age and ceased at P7.

### SC-to-HC transdifferentiation and p120ctn disorganization decrease from cochlear apex to base

To determine whether differentiated cochlear SCs could only transdifferentiate in specific regions, we cultured cochleae from P0 mice from the apex to the base in the presence of DAPT for 4 days (Figures 4G,H). DMSO treatment served as the control (Figures 4G,H). There were significant increases in Myo7a^+^ HCs and marked decreases in Prox1^+^ SCs from the apex to the mid-base in DAPT-treated cochleae compared with DMSO-treated control cochleae (Figures 4G,H; *p* < 0.01, respectively). In the DAPT-treated group, there were two-fold more Myo7a^+^ HCs in the apex, 1.8-fold more Myo7a^+^ HCs in the mid-apex and 1.4-fold more Myo7a^+^ HCs in the mid-base compared with the vehicle control (Figure 4G). The number of Prox1^+^ SCs in the DAPT-treated cochleae decreased 79% in the apex, 74% in the mid-apex, and 47% in the mid-base compared with the number of SCs in the matched regions of the vehicle controls (Figure 4H). In contrast, we did not observe significant differences in the base of the cochlea between DAPT-treated samples and controls in terms of the number of Myo7a^+^ HCs or Prox1^+^ SCs (Figures 4G,H; *p* > 0.05, respectively). Our results demonstrate that Notch inhibition exhibits a region-specific capacity to induce HC generation, with the highest activity in the apex and decreased activity toward the base.

**Figure 6:**
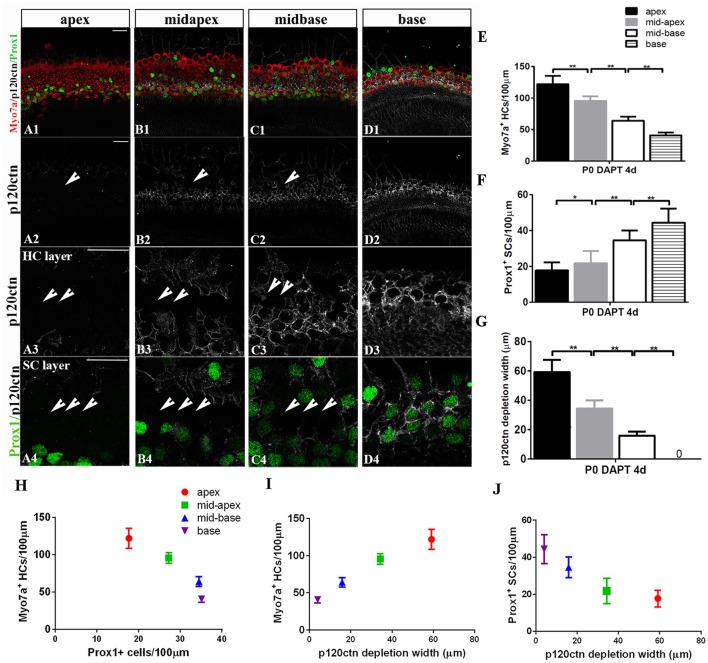
p120ctn depletion and SC-to-HC conversion gradually decrease from the apex to the base. **(A–D)** The apex, mid-apex, mid-base, and base of P0 cochleae were treated with DAPT and immunolabeled with Myo7a (red), Prox1 (green), and p120ctn (white). p120ctn was imaged with the same confocal intensity from the apex to the base. **(A2–D2)** Images show p120ctn expression from the apex to the base. p120ctn deletion occurred from the apex to the mid-base of the sensory epithelium (short arrows in **A2–C2**). **(A3–D3)** Higher magnification of p120ctn in the HC layers. Loss of p120ctn was detected in the HC layers (short arrows in **A3–C3**). **(A4–D4)** Higher magnification of p120ctn and Prox1^+^ SCs in the SC layers. p120ctn was depleted and Prox1^+^ SCs disappeared in the sensory region. The depleted areas decreased in a gradient from the apex to the mid-base (short arrows in **A4–C4**). Some scattered SCs retained p120ctn and did not exhibit any phenotype conversion. **(E)** Number of Myo7a^+^ HCs/100 μm in the sensory region. **(F)** Number of Prox1^+^ SCs/100 μm in the sensory region. **(G)** Widths of p120ctn depletion in the sensory region. **(H)** Scatterplot of the number of Myo7a^+^ HCs/100 μm (*y*-axis) and Prox1^+^ SCs/100 μm (*x*-axis) from the apex to the base of P0 cochleae. A higher number of retained Prox1^+^ SCs was associated with the generation of fewer Myo7a^+^ HCs. **(I,J)** Schematic diagram of Myo7a^+^ cells/100 μm (**I**, *y*-axis), Prox1^+^ cells/100 μm (**J**, *y*-axis) and p120 depletion widths (*x*-axis) from the apex to the base. A greater depletion of p120ctn was associated with the generation of more Myo7a^+^ HCs and the preservation of fewer Prox1^+^ SCs. The data in **(E–J)** are presented as the means ± SEMs. ^*^p < 0.05, ^**^p < 0.01. The scale bars represent 25 μm.

The observation of region-specific HC regeneration raises the question of whether E-cadherin/p120ctn disorganization could also differ between regions. Therefore, we scanned the apex to the base with the same confocal intensity and measured the length of the area of p120ctn depletion (Figures 6A–D). After 4 days of DAPT treatments in the apex, patches of junctional p120ctn were depleted along with an increase in Myo7a^+^ HCs and a decrease in Prox1^+^ SCs extending from the apical surface to the SC layer (Figure 6A, short arrows). Cochlear SCs in the mid-apex and the mid-base also exhibited reduced p120ctn and were converted into HCs in the presence of DAPT (Figures 6B,C, short arrows). However, the depleted regions in the sensory epithelium of the middle turn were discontinuous and sporadic (Figures 6B,C, short arrows). The length of the depleted region was significantly shorter in the mid-apex compared with the apex (34.35 ± 5.57 vs. 59.12 ± 8.39 μm, *p* < 0.01; *n* = 3 and 5, respectively) and in the mid-base compared with the mid-apex (15.83 ± 2.89 vs. 34.35 ± 5.57 μm, *p* < 0.01; *n* = 3 and 3, respectively; Figure 6G), indicating that disruption of p120ctn localization also differs regionally. Some scattered SCs retained p120ctn and failed to convert into HCs (Figures 6B4,C4). Therefore, we analyzed the association of p120ctn depletion with the number of Myo7a^+^ HCs and Prox1^+^ SCs from the apex to the base. Our results revealed that increased severity of p120ctn depletion was associated with increased generation of Myo7a^+^ HCs and preservation of fewer Prox1^+^ SCs (Figures 6H–J). Thus, the SC-to-HC conversion capacity and E-cadherin/p120ctn disorganization were robust in the apex and decreased toward the base in a synchronous manner.

### Longer DAPT treatments increase the amount of SC-to-HC conversion and p120ctn depletion in the apex and mid-apex turns

**Figure 7:**
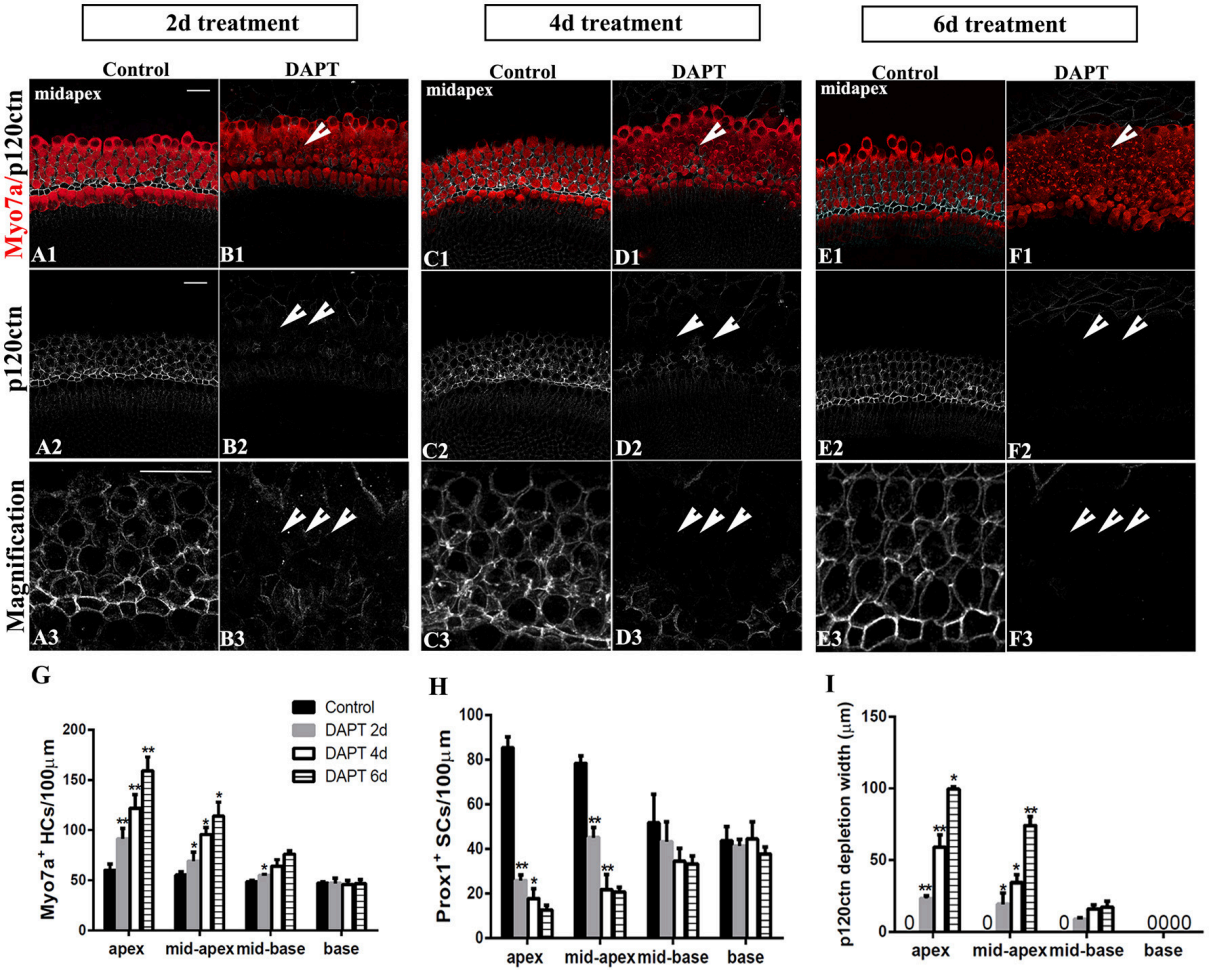
Longer DAPT treatments promote p120ctn/Prox1^+^ SC depletion and SC-to-HC conversion in the apex and mid-apex of P0 cochleae. **(A–F)** Mid-apexes harvested from P0 mice were treated with DMSO or DAPT for 2, 4, or 6 days**.** Myo7a (red), Prox1 (green), and p120ctn (white). **(A2–F2)** p120ctn expression in the apex for 2–6 days. **(A3–F3)** Higher magnification of p120ctn. A longer DAPT treatment was associated with greater p120ctn depletion. The short arrows in **(B,D,F)** indicate the regions with p120 depletion. **(G–I)** Comparison and quantification of Myo7a^+^ HCs, Prox1^+^ SCs, and p120 depletion widths in the mid-apexes of P0 mice treated with DAPT for 2, 4, and 6 days. The data in **(G–I)** are presented as the means ± SEMs. ^*^p < 0.05. ^**^p < 0.01. The scale bars represent 25 μm.

We further investigated the effects of longer DAPT treatments on explanted cochleae (Figure 7). For P0 cochleae, we reduced the period of the DAPT treatment from 4 days to 2 days or extended it to 6 days. In the apexes or mid-apexes of P0 cochleae treated with DAPT for 4 days, the number of Myo7a^+^ HCs significantly increased and the number of Prox1^+^ cells significantly decreased, and the region of p120ctn depletion was much wider compared with that of those treated with DAPT for 2 days (Figures 7G–I; *p* < 0.05, respectively). In the apexes or mid-apexes of P0 cochleae treated with DAPT for 6 days, there were significant increases in the Myo7a^+^ HCs numbers and p120ctn depletion and a prominent decrease in the number of Prox1^+^ cells compared with those treated with DAPT for 4 days (Figures 7G–I; *p* < 0.05, respectively). In the mid-basal or basal turn, however, there was no significant difference in the number of Myo7a^+^ HCs or Prox1^+^ SCs, and there was no difference in p120ctn depletion between P0 cochleae treated with DAPT for 2 days and those treated with DAPT for 4 days or between P0 cochleae treated with DAPT for 4 days and those treated with DAPT for 6 days (Figures 7G–I; *p* > 0.05, respectively). The above results indicate that longer DAPT treatments led to more SC-to-HC conversion and p120ctn depletion in the apex and mid-apex turns of P0 cochleae.

## Discussion

Many studies have investigated differences in the inner ears of mammals and non-mammals to understand the absence of spontaneous HC replacement in mammals (Hackett et al., 2002; Davies et al., 2007; Meyers and Corwin, 2007; Burns et al., 2008, 2013; Collado et al., 2011b; Burns and Corwin, 2014). Fundamental differences in the cellular structure of E-cadherin and the F-actin belts account for the different responses in HC generation in the vestibular system of mammals and non-mammals (Hackett et al., 2002; Burns et al., 2008, 2013; Collado et al., 2011b; Burns and Corwin, 2014). However, little is known regarding whether the SC-to-HC conversion capacity is linked to E-cadherin localization in mammalian cochlea. Our experiments demonstrated that the region-specific and age-dependent SC-to-HC conversion competency in the mouse cochleae was strongly correlated with the postnatal accumulation of E-cadherin/p120ctn at cell junctions. An apparent E-cadherin/p120ctn disruption occurred along with SC-to-HC conversion following DAPT induction. Our findings indicate the potential importance of junctional E-cadherin/p120ctn in preventing SC-to-HC conversion in the mammalian cochleae.

### E-cadherin/p120ctn reinforcement is correlated with the absence of HC regeneration in the postnatal cochleae

E-cadherin and its cytoplasmic partners, namely, catenins, mediate cell-cell adhesion, providing signals for contact inhibition (Steinberg, 2007; Ishiyama et al., 2010; Van den Bossche et al., 2012). The p120ctn protein is responsible for stabilizing cadherin-catenin complexes at the cell surface via its juxtamembrane domain (JMD) (Thoreson and Reynolds, 2002; Davis et al., 2003; Xiao et al., 2003; Reynolds, 2007), which regulates cadherin clustering and adhesive strengthening (Yap et al., 1998), and suppressing cell motility (Chen et al., 1997). Therefore, disruption of E-cadherin and its association with p120ctn destabilizes cell-cell adhesions and allows cancer cells to invade and metastasize (Thoreson and Reynolds, 2002). In our study, E-cadherin internalization and p120ctn depletion in the OHC region occurred along with a significant increase in the number of Myo7a^+^ HCs at the expense of Prox1^+^ SCs (Figure 3). In addition, some scattered SCs retained their junctional p120ctn after DAPT treatment. Such p120ctn-expressing SCs exhibited no detectable changes in their phenotype. Additionally, the SCs in control cochleae were surrounded by E-cadherin/p120ctn in the cytomembrane and did not undergo any SC-to-HC phenotypic conversion.

We could not determine the sequence of E-cadherin/p120ctn disruption and HC regeneration following Notch inhibition. It is possible that Notch inhibition first leads to disorganization of E-cadherin/p120ctn complexes and then induces the SC-to-HC conversion. It is also possible that Notch inhibition first leads to SC-to-HC conversion and then to E-Cadherin/p120 disorganization. The Notch signaling pathway is known to be required to maintain a mosaic distribution pattern of SCs and HCs through a lateral inhibition effect by mediating the Jagged1/Notch receptor. Loss of Notch signaling generates supernumerary HCs at the expense of SCs (Kiernan et al., 2005; Hayashi et al., 2008; Mizutari et al., 2013; Li et al., 2015; Ni et al., 2016a; Waqas et al., 2016b). Notch signaling might also mediate junction stability by localizing E-cadherin and its associated catenins to SC/HC contacts.

In our study, we did not detect any differences in E-cadherin or p120ctn protein or mRNA levels between DAPT- and DMSO-treated cochleae (Figure 3) according to the Western blot and qPCR results. Similar results have been observed in cell culture experiments (Swaminathan and Cartwright, 2012). Following tyrosine phosphorylation of the E-cadherin/p120ctn complex, E-cadherin/p120ctn complexes were removed from the cytomembrane, with E-cadherin diffusely distributed throughout the cytoplasm. However, there were no differences in the total levels of either E-cadherin or p120ctn compared with the controls (Swaminathan and Cartwright, 2012).

Previously, p120ctn was reported to be a gatekeeper of E-cadherin turnover via its ability to bind to the intracellular domains of cadherins (Thoreson and Reynolds, 2002; Davis et al., 2003; Xiao et al., 2003; Perez-Moreno et al., 2006; Miyashita and Ozawa, 2007; Reynolds, 2007). Uncoupling of the p120ctn-JMD interaction or knockdown of p120ctn leads to significant reductions in membrane-localized cadherins *in vitro* and *in vivo* (Miyashita and Ozawa, 2007; Reynolds, 2007; Chacon-Heszele et al., 2012). Therefore, in our experiment, disruption of p120ctn localization could represent the disintegration of E-cadherin/p120ctn complexes. Therefore, we used p120ctn to further investigate the correlation between E-cadherin/p120ctn localization and region- or age-dependent SC-to-HC conversion.

### Age-dependent E-cadherin/p120ctn disruption and SC-to-HC phenotype conversion in the postnatal cochleae

We found that a precipitous age-dependent decline in the ability of DAPT induces supporting cell trans-differentiation into hair cells, which is consistent with previous studies (Kelly et al., 2012; Liu et al., 2012; Cox et al., 2014; Shi et al., 2014). By 7 days after birth, the organ of Corti is essentially unresponsive to Notch inhibition in culture. This type of age-related SC-to-HC conversion capacity is linked to the accumulation of F-actin and E-cadherin in postnatal utricles (Hackett et al., 2002; Burns et al., 2008, 2013; Collado et al., 2011b; Burns and Corwin, 2014). We found that junctional p120ctn in the sensory epithelia of P0, P3, and P7 cochleae exhibited distinct differences in response to Notch inhibition. SCs from the apex to the mid-base of P0 mice and from the apex of P3 mice were converted into HCs along with p120ctn disruption in the presence of DAPT. However, neither SC-to-HC conversion nor p120ctn depletion was observed in the mid-apex to the base of P3 mice or in any area of the cochlea of P7 mice. The SCs that retained p120ctn did not exhibit any phenotype conversion (Figure 5). Additionally, areas of p120ctn depletion in the apex of P3 mice were notably smaller than those in the apex of P0 mice. The decline in the capacity of SC-to-HC differentiation occurred in parallel with the accumulation of junctional E-cadherin/p120ctn in postnatal SCs undergoing maturation (Figure 1), supporting the hypothesis that age-dependent SC-to-HC differentiation is correlated to postnatal localization of junctional E-cadherin/p120ctn in the mammalian cochlea.

### Region-specific HC generation is linked to postnatal E-cadherin/p120ctn accumulation in the mouse cochlea

Studies of the vestibular system have previously demonstrated that the SCs of differentiated utricles exhibit region-dependent limitations in their capacity to change phenotype (Burns et al., 2008; Collado et al., 2011b; Burns and Corwin, 2014), which is strongly correlated with differences in E-cadherin organization between striolar and extrastriolar regions (Collado et al., 2011b). Similar to the vestibular system, postnatal E-cadherin accumulation in mice cochleae is involved in region-specific SC-to-HC differentiation. We found that the SC-to-HC conversion capacity in cochleae gradually decreased from the apex to the base in response to Notch inhibition, which is consistent with previous studies (Cox et al., 2014; Li et al., 2015; Maass et al., 2015; Ni et al., 2016a; Waqas et al., 2016a). SCs in the apical turn have a higher capacity for SC differentiation and HC generation than those in the basal turn (Cox et al., 2014; Li et al., 2015; Maass et al., 2015; Ni et al., 2016a; Waqas et al., 2016a). Studies have shown that either expression of Lgr5 progenitors (Waqas et al., 2016a) or down-regulation of Notch receptors, ligands and effectors (Maass et al., 2015) in a basal-apical gradient leads to region-specific HC regeneration. In our study, we demonstrated that SCs in the basal turn of P0 mice were preserved and maintained their junctional p120ctn even after longer DAPT treatments, whereas the number of SCs significantly decreased in the apex and the mid-apex, where p120ctn was extensively depleted (Figure 6). In addition, longer DAPT treatments induced more SC-to-HC conversion and p120ctn depletion in the apex and mid-apex turns but did not induce changes in the mid-basal or basal turns, indicating that the HC generative potential continues for longer in the apex than in the base of the cochlea. Both a decreased response to Notch inhibition and p120ctn depletion progress in a basal-apical gradient along the organ of Corti, consistent with the gradient of cellular differentiation in the cochlea, which starts at the base at approximately E14 and progresses toward the apex with a delay of 3 days (Chen and Segil, 1999; Chen et al., 2002). It would be interesting to explore whether prolonged DAPT treatment or prolonged culture following DAPT treatment could lead to more SC-to-HC conversions in both the apex and mid-apex regions. The findings here highlight the potential importance of junctional E-cadherin/p120ctn as a regulator of cellular phenotype stability and maturation of the auditory epithelium.

## Conclusion

In summary, our study demonstrated that postnatal region-specific and age-dependent SC-to-HC conversion is strongly correlated with E-cadherin/p120ctn complexes in the mammalian cochlea. These findings provide new insights for determining the factors responsible for terminating HC regeneration in the mammalian cochleae. In future experiments, it would be of great interest to explore the detailed molecular mechanism of E-cadherin/p120ctn disruption following Notch inhibition. In addition, overexpression of E-cadherin and/or p120ctn in neonatal mice cochleae or conditional knock out of E-cadherin and/or p120ctn in adult mice provide a better understanding of their roles in SC-to-HC conversion. Moreover, it would be worth exploring whether F-actin belts or other members of the catenin family, such as α-, β-, or γ-catenin, regulate the SC-to-HC differentiation capacities in different species.

## Author contributions

WL, DR, PC, FC, and JY: Designed the experiments; WL, XW, RM, NC, and YG: Performed the experiments; WL and JY: Wrote and revised the paper; PC, FC, DR, NC, and JY: Provided financial support for the project.

### Conflict of interest statement

The authors declare that the research was conducted in the absence of any commercial or financial relationships that could be construed as a potential conflict of interest.
